# Larval description and chaetotaxic analysis of *Dineutus sinuosipennis* Laporte, 1840, with a key for the identification of larvae of the tribe Dineutini (Coleoptera, Gyrinidae)

**DOI:** 10.3897/zookeys.718.20726

**Published:** 2017-12-04

**Authors:** Mariano C. Michat, Grey T. Gustafson, Johannes Bergsten

**Affiliations:** 1 University of Buenos Aires, Faculty of Exact and Natural Sciences, Department of Biodiversity and Experimental Biology, Laboratory of Entomology, Buenos Aires, Argentina; 2 CONICET–University of Buenos Aires, Institute of Biodiversity and Experimental and Applied Biology, Buenos Aires, Argentina; 3 Department of Ecology and Evolutionary Biology, University of Kansas, Lawrence, KS 66045, USA; 4 Department of Zoology, Swedish Museum of Natural History, Stockholm, Sweden

**Keywords:** Adephaga, whirligig beetles, larva, sensilla, DNA-based association

## Abstract

The larvae of the Malagasy whirligig beetle *Dineutus sinuosipennis* Laporte, 1840, identified using DNA sequence data, are described and illustrated for the first time, including detailed morphometric and chaetotaxic analyses of selected structures and a description of larval habitat. Larvae of the genus *Dineutus* Macleay, 1825 are diagnosed, and a key to identify the genera of the tribe Dineutini is presented. Larvae of *Dineutus* exhibit the characters traditionally recognized as autapomorphies of the Gyrinidae: body less sclerotized, egg bursters located on the parietal, one additional sensorial plate on the third antennomere, cardo and lacinia well developed, prementum completely divided, abdominal tracheal gills, and four terminal hooks on the pygopod. They also share with larvae of the other Dineutini genera these putative synapomorphies: numerous minute pore-like additional structures on the ultimate maxillary and labial palpomeres, coxal primary seta CO12 inserted submedially, and trochanteral primary seta TR2 absent. Larvae of *Dineutus* can be distinguished from those of other known genera of Dineutini by the posterior margin of the lacinia not dentate, tracheal gills plumose, parietal seta PA5 inserted relatively far from setae PA7–9, mandibular pores MNb and MNc inserted relatively far from each other, and tarsal seta TA1 inserted submedially.

## Introduction

The genus *Dineutus* Macleay, 1825 comprises 92 relatively large-sized species and has a near global distribution, being absent from Europe and, notably, from South America (Gustafson and Miller 2017). The species are common in both lotic and lentic aquatic environments including ponds, lakes, rivers, and streams, although most individuals are found in slower parts of streams and rivers (Gustafson and Miller 2015). It is included in the tribe Dineutini together with the extant genera *Enhydrus* Laporte, 1835, *Macrogyrus* Régimbart, 1882 and *Porrorhynchus* Laporte, 1835, and one extinct genus (Gustafson and Miller 2017). Numerous subgenera were erected within *Dineutus*, although presently only three are recognized: *Dineutus* s.str., *Rhomborhynchus* Ochs, 1926, and *Cyclous* Dejean, 1833, the largest subgenus, to which the species described here belongs (Gustafson and Miller 2017). *Dineutus sinuosipennis* Laporte, 1840 is endemic to Madagascar and the Comoro Islands (Brinck 1955). On Madagascar, it is a common and widespread species in slow-flowing, running waters. It can tolerate substantial anthropogenic disturbance, for instance it is often found in man-made water canals along rice paddy fields.

Larval morphology of *Dineutus* is poorly known. Larvae of a vast majority of species are unknown, and the few described ones are insufficiently documented. Nowrojee (1912) shortly described the mature larva of *D. unidentatus* Aubé, 1838, including a habitus drawing. Wilson (1923) studied the three larval instars of *D. assimilis* Kirby, 1937, focusing on the third instar for the description and illustrations, which to date remains the most detailed morphological treatment of a larva of the genus. Hatch (1927) provided an unillustrated key to separate the first instars of four species. Brief descriptions and comments, including some drawings, of a few *Dineutus* larvae were provided by Bertrand (summarized in Bertrand 1963). An unidentified species was partially examined for chaetotaxy by Archangelsky and Michat (2007), although lacking illustrations and a formal description of the chaetotaxy pattern. Images of the larvae of three species were provided by Istock (1967), without information as to which instar they belong, although their size suggests third instar. Other descriptions or treatments of *Dineutus* larvae are lacking in the literature, with the exception of some drawings of the head of *D. discolor* Aubé, 1838 in the context of phylogenetic studies (Beutel 1993, Beutel and Roughley 1994). A detailed morphological description, including chaetotaxic analysis, of all larval instars of this genus is lacking.

In recent years, several descriptions of gyrinid larvae were published, emphasizing not only general morphology but also including chaetotaxic analyses in the following genera and subgenera: Macrogyrus (Andogyrus) Ochs, 1924 (Archangelsky and Michat 2007), *Gyrinus* Müller, 1764 (Michat et al. 2010), Macrogyrus (Macrogyrus) (Michat and Gustafson 2016), and *Enhydrus* (Michat et al. 2016). A system of nomenclature for the primary sensilla of larvae of the family Gyrinidae has not been fully developed so far, mainly because larvae of several genera are still unknown. However, although likely subject to improvement based on the discovery of more gyrinid larvae, these studies provide a descriptive template to which larvae of additional genera can be incorporated.

In the present paper, a detailed description of all larval instars of the genus *Dineutus* is provided, including, for the first time, morphometric and chaetotaxic analyses of the cephalic capsule, head appendages and legs of *D. sinuosipennis*. Comparisons with other gyrinid genera for which the larvae have been described in detail are also provided, and an identification key to separate all instars of the known genera of the tribe Dineutini is presented. Despite adults being collected readily by the hundreds, larvae of many gyrinid species remain frustratingly elusive. For this reason, we also provide a description of the precise habitat where larvae of *D. sinuosipennis* were collected to aid future collection.

## Material and methods

The descriptions provided in this paper are based on two specimens of instar I, three of instar II and three of instar III collected in Madagascar, at the locality described below.

### Identification of larvae and molecular procedures

There are three species of *Dineutus* known from Madagascar: *D. proximus* Aubé, 1838, *D. sinuosipennis* and *D. subspinosus* (Klug, 1834). Only adults of *D. sinuosipennis* were found at the locality where the larvae were collected, but for unambiguous association we extracted DNA from four larvae of instar III and sequenced mitochondrial cytochrome c oxidase subunit I (COI) using the primers ‘Jerry’ (Simon et al. 1994) and ‘Pat Dyt’ (Isambert et al. 2011). The legs of one side were removed for lysis and DNA extraction using Qiagen Blood and Tissue kit following standard protocol; remaining body was retained as vouchers (Catalogue numbers: NHRS-JLKB000001600 – NHRS-JLKB000001603).

PCRs were set up in 25 ul reactions using Illustra Hot start mix RTG (GE Healthcare, Little Chalfont, Buckinghamshire, UK), 1 ul each of the Primers (10 uM), 2ul of DNA template and 21 ul water. Thermal cycling profile included the following steps: 95 °C for 5 min followed by 40 cycles of 95 °C for 30 s, 50 °C for 30 s and 72 °C for 60 s, and finally 72 °C for 8 min. Products were cleaned with the ExoFast method, using a combination of Exonuclease 1 and FastAP (ThermoFisher Scientific, Waltham, MA, USA). Cleaned products were sequenced using Big Dye version 3.1 on an ABI 3130XL Genetic Analyzer. The molecular lab work was carried out by the Centre for Genetic Identification (CGI), Swedish Museum of Natural History, Stockholm, Sweden.

Forward and reverse reads were combined into contigs in Sequencher (version 4) and primer regions were removed. The sequences were trimmed to the 740bp length fragment used by Isambert et al. (2011). All full-length fragments of the three known Madagascan species of *Dineutus* were downloaded from Genbank and combined with the new data. New sequences were submitted to Genbank under accession codes MG489880–MG489883. For the 80 terminal taxa, 740 bp alignment contained no gaps and matrix completeness was 100%. For a tree-based association of larvae with adults we used Beast, with the input xml-file set up in Beauti (both v. 1.8.4). We used a strict clock model to root the tree and a HKY+G substitution model, with codon positions partitioned to allow them separate overall rates but with tree, clock and substitution models linked. We used a constant-size coalescent tree prior as most branching events in the tree are within species coalescence events. Other prior settings were left as default. Two separate runs, each with 10M generations (sampling frequency 2000) were run, 25% burn-in removed from each run before combined in LogCombiner and a maximum clade credibility tree calculated with TreeAnnotator. Convergence between runs and ESS values (all >200) were evaluated in Tracer v. 1.6. We used the Generalized Mixed Yule Coalescent (GMYC) method (Fujisawa and Barraclough 2013) to test for conspecificity between larvae and identified adults. The GMYC analysis using the ultrametric tree from Beast applied the single threshold method and was run in R with the Splits package.

### Methods for the study of larvae

The larvae were cleared in lactic acid, dissected, and mounted on glass slides in polyvinyl-lacto-glycerol. Microscopic examination at magnifications up to 1,000× and drawings were made using an Olympus CX31 (Olympus Corporation, Tokyo, Japan) compound microscope equipped with a camera lucida. Drawings were scanned and digitally inked using a Genius PenSketch tablet (KYE Corporation, Taipei, Taiwan). The material is held in the collection of the senior author (Laboratory of Entomology, Buenos Aires University, Argentina).

### Morphometric analysis

We employed the terms used in previous papers dealing with the larval morphology of Gyrinidae (Archangelsky and Michat 2007; Michat et al. 2010, 2016; Michat and Gustafson 2016). The following measurements were taken (with abbreviations shown in parentheses). Total length (excluding terminal tracheal gills) (TL); maximum width (excluding tracheal gills) (MW); head length (HL) (total head length including the frontoclypeus, measured medially along the epicranial stem); maximum head width (HW); length of frontoclypeus (from anterior margin to the joint of frontal and coronal sutures) (FRL); occipital foramen width (maximum width measured along dorsal margin of occipital foramen) (OCW); coronal suture length (COL); length of mandible (MNL) (measured from laterobasal angle to apex); width of mandible (MNW) (maximum width measured at base); length of maxillary palpifer (PPF); length of galea (GA). Length of antenna (A), maxillary (MP) and labial (LP) palpi were derived by adding the lengths of the individual segments; each segment is denoted by the corresponding letter(s) followed by a number (e.g., A1, first antennomere). The maxillary palpus was considered as being composed of three segments united to the stipes through a palpifer (Archangelsky and Michat 2007). Length of leg, including the longest claw (CL), was derived by adding the lengths of the individual segments; each leg is denoted by the letter L followed by a number (e.g., L1, prothoracic leg); the length of trochanter includes only the proximal portion, considered from the base to the beginning of the femur; the leg was considered as being composed of six segments (Lawrence 1991). Length of terminal hooks of abdominal segment X, separated in medial hook (MH) and lateral hook (LH). These measurements were used to calculate several ratios that characterize body shape.

### Chaetotaxic analysis

Primary setae and pores were distinguished in the cephalic capsule, head appendages and legs. Sensilla were coded by two capital letters, in most cases corresponding to the first two letters of the name of the structure on which they are located, and a number (setae) or a lower case letter (pores). The following abbreviations were used: AN, antenna; CO, coxa; FE, femur; FR, frontoclypeus; LA, labium; MN, mandible; MX, maxilla; PA, parietal; PT, pretarsus; TA, tarsus; TI, tibia; TR, trochanter. Setae and pores present in the first-instar larva of *D. sinuosipennis* were labeled by comparison with previous papers dealing with the primary chaetotaxy of members of the family Gyrinidae (Archangelsky and Michat 2007, Michat et al. 2010, 2016; Michat and Gustafson 2016). Homologies were recognized using the criterion of similarity of position (Wiley 1981). Setae located at the apices of the maxillary and labial palpi were extremely difficult to distinguish due to their position and small size. Accordingly, they are not well represented in the drawings.

## Results

### Molecular results

**Figure 1.**
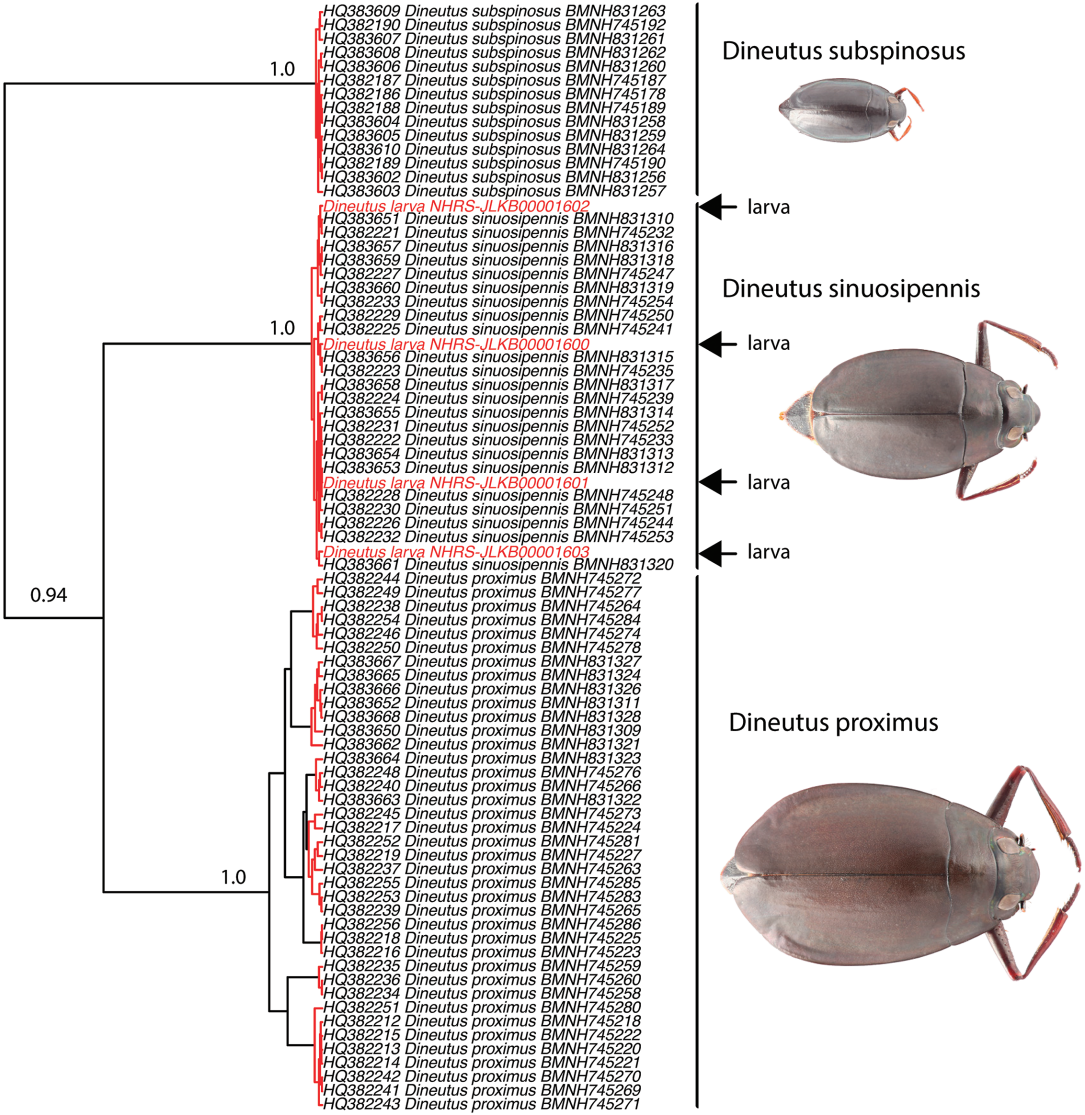
Ultrametric COI gene tree from Beast used to test larvae conspecificity with identified adults using GMYC. Red branches are intraspecific coalecence events, black branches represent divergence between separately evolving lineages. Numbers at nodes are posterior probability values from Beast (only given for species nodes). Sequences from the four larvae are conspecific with adults of *Dineutus sinuosipennis* but heterospecific to adults of *D. proximus* and *D. subspinosus*, according to the GMYC results.

The 740 bp COI fragment matching the dataset of Isambert et al. (2011) was successfully amplified for all four larvae. The ultrametric gene tree from Beast recovered the four larvae in a cluster together with adults identified as *D. sinuosipennis* (Fig. 1). The maximum genetic distance, calculated as uncorrected p-distances, between a larva and its closest match among *D. sinuosipennis* was 0.41%. The intraspecific genetic variation within *D. sinuosipennis* was 0.59% on average (min-max: 0–1.49%). Likewise, the intraspecific variation for *D. subspinosus* was small (0–0.81%, mean 0.37%). However, *D. subspinosus* is the only Madagascan gyrinid that also occurs on mainland Africa, and a larger geographic sampling would likely yield a larger intraspecific distance (Bergsten et al. 2012). Intraspecific variation in *D. proximus*, by contrast, was larger (0–5.27%, mean 3.19%). Interspecific genetic distances between all three species were much larger (9.5–13.1%), including those between the larvae and any of the other two species. The GMYC analysis recovered the four larvae as conspecific with the adults identified as *D. sinuosipennis* in the maximum likelihood solution (Fig. 1). As has been reported before (Isambert et al. 2011), *D. proximus* represented multiple separately evolving lineages in the maximum likelihood solution from the GMYC analysis, a genetic structure that also has a geographic correlation.

### Diagnosis of larvae of *Dineutus* Macleay, 1825

**Figures 2–3.**
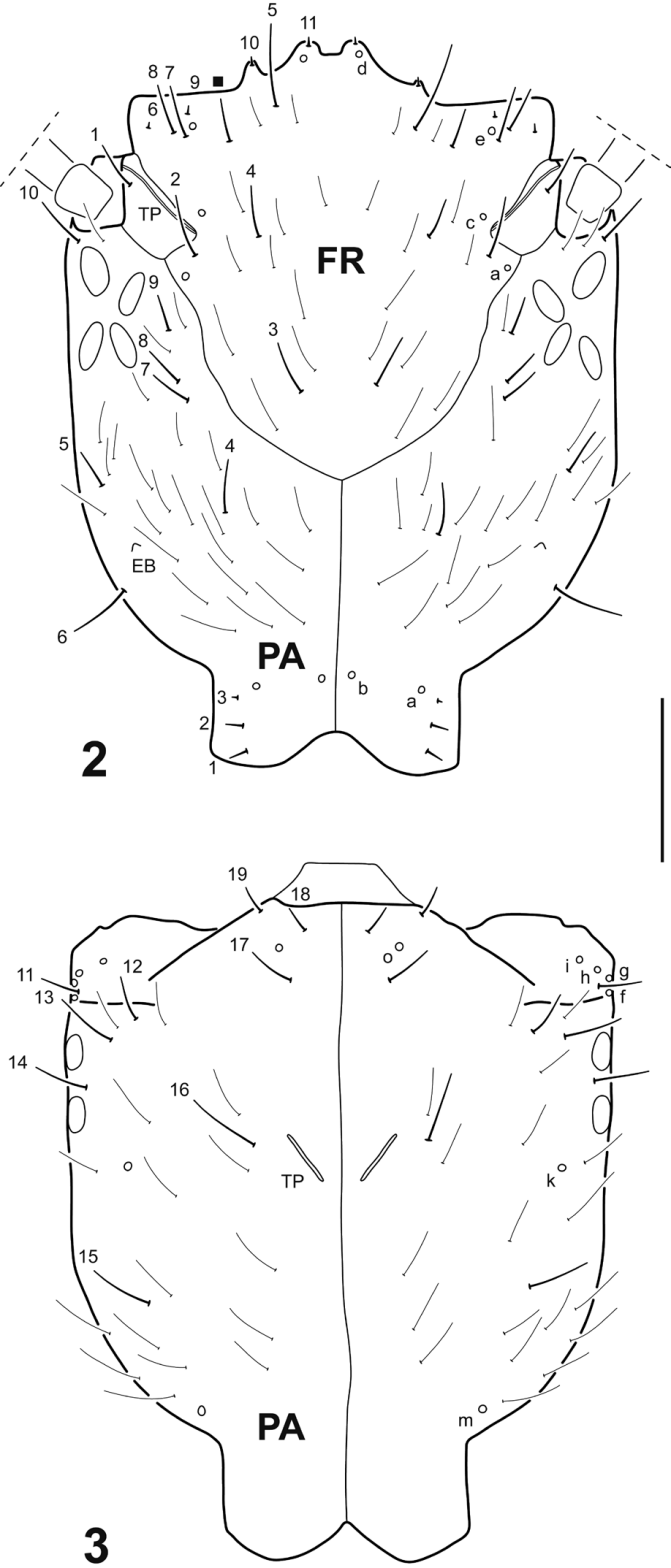
*Dineutus sinuosipennis* Laporte, 1840, instar I. **2** Cephalic capsule, dorsal view **3** Cephalic capsule, ventral view. Numbers and lowercase letters indicate primary setae and pores, respectively. Conspicuous additional seta on frontoclypeus indicated by a solid square. Inconspicuous additional setae not labeled. EB: egg burster; FR: frontoclypeus; PA: parietal; TP: tentorial pit. Scale bar: 0.20 mm.

Cephalic capsule constricted at level of occipital region (Figs 2–3, 16); occipital suture absent in instar I (Fig. 2), absent or weakly delimited in instars II and III (Fig. 16); coronal suture relatively long (Fig. 2); medial lobe of frontoclypeus well produced forward, clearly asymmetrical in instar I (sometimes also in instars II and III), with four relatively well defined teeth (Figs 2, 16), medial teeth sometimes not well differentiated from each other (Fig. 26); cardo subrectangular in instar I (Figs 7–8); lacinia not serrate on posterior margin, indented apically (Figs 7–8); claws lacking basoventral spinulae (Figs 11–12); tracheal gills bearing long spinulae; terminal hooks subequal in length (Figs 13–15); seta PA5 inserted relatively far from setae PA7–9 (Fig. 2); pores MNb and MNc inserted relatively far from each other (Fig. 6); mandible with additional setae (instar I) (Fig. 6); cardo with a single additional seta (instar I) (Fig. 8); pore MXg proximal (Fig. 8); maxillary palpomeres 1 and 2 and labial palpomere 1 lacking minute pore-like additional structures (Figs 7–10); prementum with one additional pore (Fig. 10); seta CO12 inserted submedially (Fig. 12); coxa lacking additional setae (Figs 11–12); seta TR2 absent (Fig. 11); seta TA1 inserted submedially (Fig. 12); coxa with secondary setae (instars II–III); abdominal segment X lacking ventral spinulae (Fig. 13).

### Description of larvae of *Dineutus sinuosipennis* Laporte, 1840

***Instar I*** (Figs 2–15)

Color. Cephalic capsule and mandibles light brown, antennae, maxillae and labium testaceous; thoracic sclerites light brown, rest of thorax and legs testaceous; abdomen testaceous except terminal hooks light brown.

Body. Elongate, parallel sided, head and pronotum strongly sclerotized, rest of thorax and abdomen soft. Measurements and ratios that characterize the body shape are shown in Table 1.

**Table 1.** Measurements and ratios for the larvae of *Dineutus sinuosipennis* Laporte, 1840.

Measure	Instar I (n = 2)	Instar II (n = 3)	Instar III (n = 3)
TL (mm)	5.50	14.80	20.10–28.30
MW (mm)	0.70	2.20	2.40–3.30
HL (mm)	0.87–0.91	1.38–1.42	2.02–2.05
HW (mm)	0.65–0.66	0.99–1.01	1.42–1.51
FRL (mm)	0.51–0.55	0.78–0.81	1.14–1.19
OCW (mm)	0.28–0.30	0.55–0.60	0.94–0.99
COL (mm)	0.36	0.59–0.60	0.84–0.88
HL/HW	1.34–1.37	1.37–1.43	1.34–1.43
HW/OCW	2.20–2.30	1.64–1.79	1.49–1.53
COL/HL	0.39–0.41	0.43	0.41–0.44
FRL/HL	0.59–0.61	0.57	0.56–0.59
A/HW	1.07	0.97–1.01	0.83–0.90
A1/A3	0.24–0.25	0.24–0.29	0.28–0.30
A2/A3	0.71–0.83	1.07–1.15	1.28–1.43
A4/A3	0.96–1.04	0.80–0.88	0.73–0.74
MNL/MNW	2.94–3.21	3.05–3.12	2.85–2.93
MNL/HL	0.49–0.53	0.45–0.47	0.43
A/MP	1.34–1.36	1.22–1.40	1.25–1.28
GA/MP1	1.00–1.04	0.77–0.84	0.65–0.72
PPF/MP1	0.50–0.55	0.48–0.50	0.31–0.36
MP1/MP2	0.65–0.75	0.88–0.96	1.19–1.30
MP3/MP2	1.38–1.59	1.15–1.26	1.06–1.12
MP/LP	1.22–1.29	1.23–1.28	1.28–1.31
LP2/LP1	1.16–1.20	0.84–0.89	0.70–0.73
L3 (mm)	1.74–1.85	2.86–2.88	4.72–4.75
L3/L1	1.13–1.15	1.21	1.26–1.27
L3/L2	1.06–1.07	1.08–1.10	1.11–1.12
L3/HW	2.68–2.78	2.86–2.91	3.12–3.35
L3 (CO/FE)	1.00–1.05	1.03–1.07	1.08–1.09
L3 (TI/FE)	0.61–0.67	0.56–0.57	0.54–0.58
L3 (TA/FE)	0.90–0.92	0.78–0.84	0.66–0.70
L3 (CL/TA)	0.48–0.49	0.36–0.41	0.35
MH/LH	1.02–1.05	1.04–1.06	1.07–1.17

**Figures 4–10.**
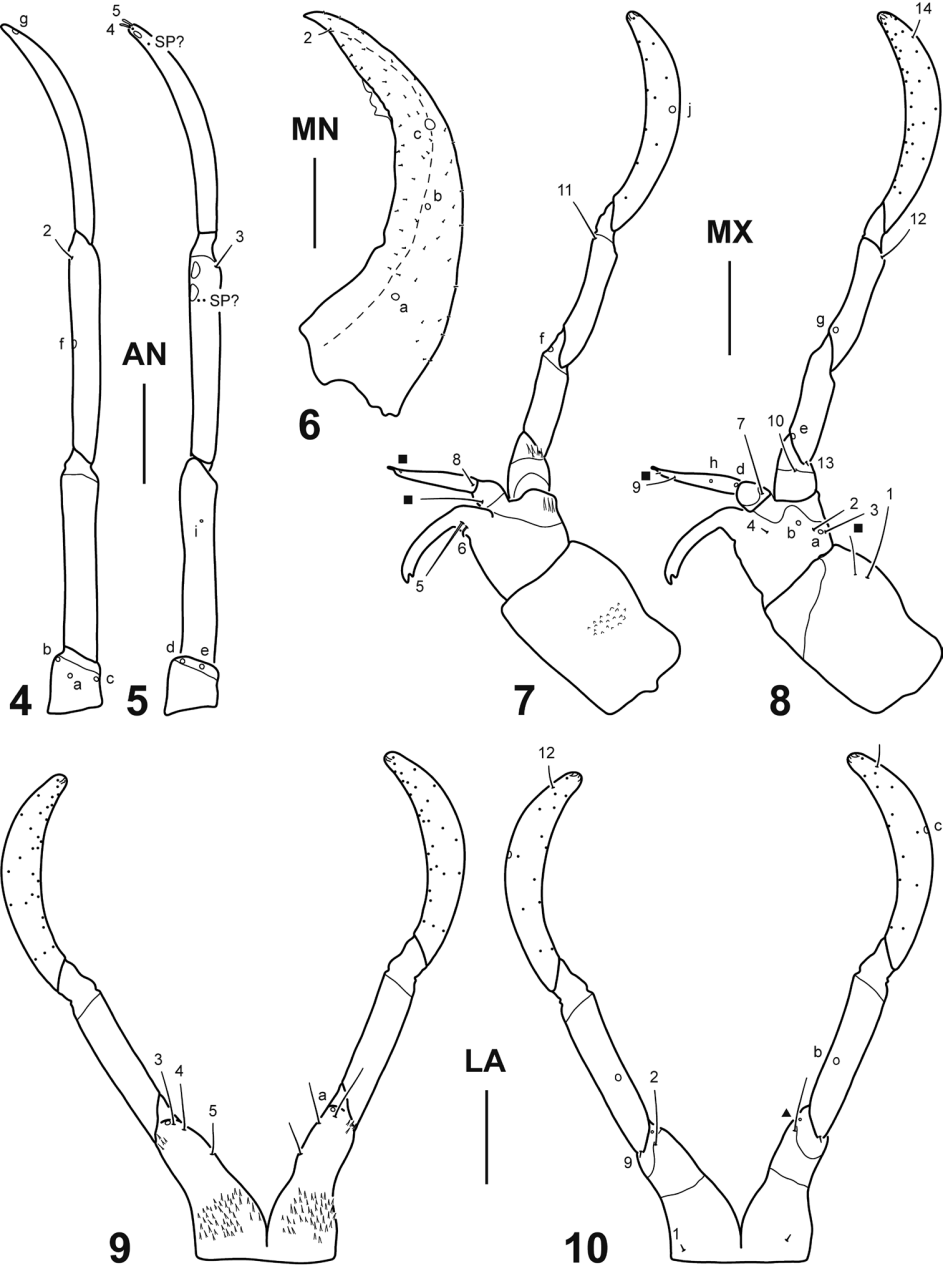
*Dineutus sinuosipennis* Laporte, 1840, instar I. **4** Right antenna, dorsal view **5** Left antenna, ventral view **6** Right mandible, dorsal view **7** Right maxilla, dorsal view **8** Left maxilla, ventral view **9** Labium, dorsal view **10** Labium, ventral view. Numbers and lowercase letters indicate primary setae and pores, respectively. Additional setae indicated by solid squares (except for minute additional setae on the mandible which are not labeled). Additional pore on prementum indicated by a solid triangle. AN: antenna; LA: labium; MN: mandible; MX: maxilla; SP: spinulae. Scale bars: 0.10 mm.

Head. *Cephalic capsule* (Figs 2–3). Subrectangular (excluding neck), longer than broad, parallel-sided with distinct narrow neck; occipital foramen emarginate both dorsally and ventrally; occipital suture absent; coronal suture relatively long; frontal sutures U-shaped, extending to antennal bases; anterior tentorial pits elongate, visible dorsally near antennal bases; posterior tentorial pits elongate, visible ventromedially; neck area rugose; FR relatively short, roughly subtriangular, anterior margin with three lobes; medial lobe well produced anteriorly, clearly asymmetrical, with four teeth, right tooth smaller and somewhat detached, the other three well developed; lateral lobes well developed, truncate, not projected beyond medial lobe; PA with egg bursters formed by a single small cuticular spine on each posterolateral surface, and six stemmata at each side, four dorsal and two ventral. *Antenna* (Figs 4–5). Moderately long, slender, slightly longer than HW, composed of four antennomeres; A1 shortest, A3 and A4 longest, subequal in length; A3 with two minute structures (probably spinulae or pores) on ventrodistal surface, and two subapical flat plates on inner margin, distal one interpreted as the sensorium (A3’) which does not protrude; A4 with a subapical flat sensorial plate on inner margin, accompanied by two minute structures (probably spinulae or pores). *Mandible* (Fig. 6). Relatively elongate, curved, broad basally, distal half projected inward, apex sharp; inner margin more or less toothed on distal third; retinaculum absent, although one of the teeth may be interpreted as that structure; mandibular channel present. *Maxilla* (Figs 7–8). Well developed, prominent; cardo strongly developed, subrectangular, bearing a group of minute spinulae on dorsal surface; stipes short, broad, subtrapezoidal, bearing a lacinia and GA on distal inner margin and a PPF on distal outer margin; lacinia well developed, slender, indented apically; GA elongate, two-segmented, basal segment shorter, distal segment longer, narrowing to apex; PPF short, palpomere-like, projected apicointernally in a subtriangular process; MP long, composed of three palpomeres separated by oblique joints; MP1 shortest, MP3 longest. *Labium* (Figs 9–10). Well developed, prominent; prementum divided longitudinally into two subcylindrical halves fused basally, each half bearing minute spinulae on dorsal surface and projected apicointernally in a subtriangular process; LP long, composed of two palpomeres separated by oblique joints; LP1 slightly shorter than LP2.

**Figures 11–16.**
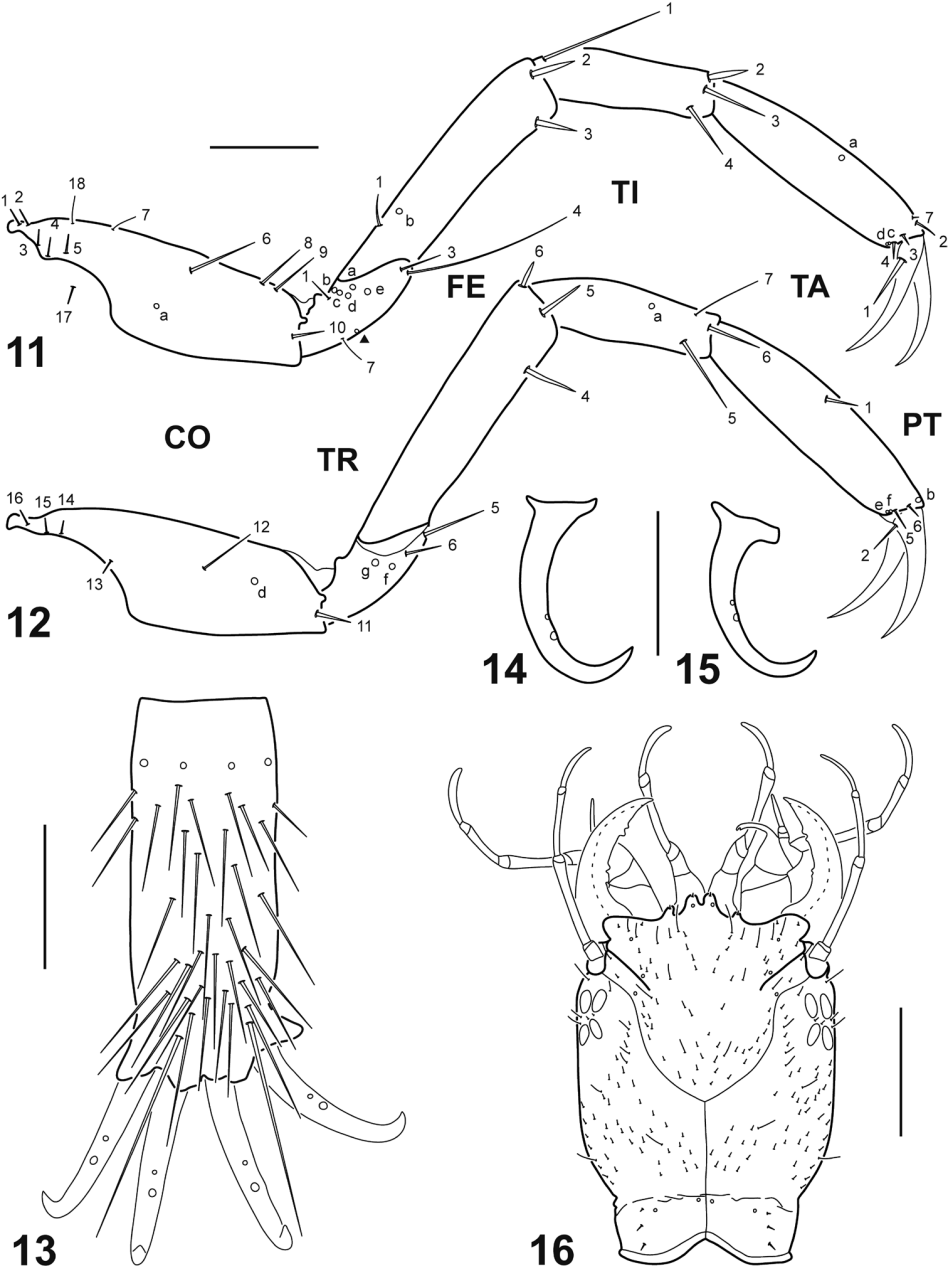
*Dineutus sinuosipennis* Laporte, 1840. **11–15** Instar I **16** Instar III **11** Left metathoracic leg, anterior view **12** Right metathoracic leg, posterior view **13** Abdominal segment X, ventral view **14** Medial hook, lateral view **15** Lateral hook, lateral view **16** Head, dorsal view. Numbers and lowercase letters indicate primary setae and pores, respectively. Additional pore on trochanter indicated by solid triangle. Sensilla on abdominal segment X not labeled. CO: coxa; FE: femur; PT: pretarsus; TA: tarsus; TI: tibia; TR: trochanter. Scale bars: 0.15 mm (**11–15**); 0.70 mm (**16**).

Thorax. Long, narrow, subcylindrical; pronotum somewhat larger that subequal meso- and metanotum; protergite well developed, covering whole segment dorsally, anterior margin truncate, lateral and posterior margins rounded; membrane between pro- and mesonotum with a single narrow transverse sclerite; both sclerites with sagittal line, lacking anterior transverse carina; meso- and metaterga lacking sclerites; ventral surface membranous except for a large subrectangular sclerite (divided in two halves by broad sagittal line) on anterior third of prothorax, and small sclerites on the regions of articulation of coxae; spiracles absent. *Legs* (Figs 11–12). Long, slender, composed of six segments; L3 longest, L1 shortest; CO elongate, robust, TR short, lacking annulus, FE, TI and TA slender, subcylindrical, PT with two long, slender, slightly curved claws, posterior claw shorter than anterior claw on L1 and L2, claws subequal in length on L3; spinulae absent.

Abdomen. Ten-segmented, long, narrow, subcylindrical, entirely membranous; segments I–VIII similar in shape, progressively smaller to apex, bearing a tracheal gill on posterolateral angle; segment IX smaller than segment VIII, bearing two tracheal gills on posterolateral angle; tracheal gills slender, plumose, those of segment IX longer than the others; all tracheal gills bearing an anterior and a posterior row of long setiform spinulae, those of segment I and, to a lesser extent of segment II with less spinulae; segment X (Fig. 13) narrowest, pygopod-like, arising on posteroventral surface of segment IX, lacking gills and ventral spinulae, bearing four strongly sclerotized terminal hooks, medial hook slightly longer than lateral hook (Figs 14–15).

Chaetotaxy. *Frontoclypeus* (Fig. 2). Medial lobe of anterior margin with two spine-like setae (FR10, FR11), one short hair-like seta (FR5), and one pore (FRd); lateral lobe of anterior margin with two minute spine-like setae (FR6, FR9), two short hair-like setae (FR7, FR8), and one pore (FRe); lateral margin with two short hair-like setae (FR1, FR2) and two pores (FRa, FRc) on distal half and one short hair-like seta (FR3) on basal half; central portion with one short hair-like seta (FR4); surface with numerous short hair-like additional setae. *Parietal* (Figs 2–3). Dorsal surface with one short hair-like seta (PA10) posterior to antennal base, a longitudinal row of three short hair-like setae (PA7, PA8, PA9) close to frontoclypeal margin, three short hair-like setae (PA4, PA5, PA6) on basal third, close to egg bursters, and three short spine-like setae (PA1, PA2, PA3) and two pores (PAa, PAb) on neck region; ventral surface with three short hair-like setae (PA17, PA18, PA19) and one pore (PAo) on anteromedial region, four short hair-like setae (PA11, PA12, PA13, PA14) and four pores (PAf, PAg, PAh, PAi) on anterolateral angle, one short hair-like seta (PA16) and one pore (PAk) at mid length, and one long hair-like seta (PA15) and one pore (PAm) on basal third; dorsal and ventral surface with numerous short hair-like additional setae (except on neck region). *Antenna* (Figs 4–5). A1 with three pores (ANa, ANb, ANc) on dorsal surface and two pores (ANd, ANe) on ventral surface; A2 with one minute pore (ANi) on ventral surface; A3 with one pore (ANf) on lateromedial region, one short hair-like seta (AN2) on dorsodistal portion and one short hair-like seta (AN3) on ventrodistal portion; A4 with one pore (ANg) on dorsodistal portion and two minute spine-like setae (AN4, AN5) at apex. *Mandible* (Fig. 6). Dorsal surface with one pore (MNa) on basal fourth, two pores (MNb, MNc) at about mid length, and one short hair-like setae (MN2) near tip; dorsal and lateral surfaces with numerous minute additional setae; we were unable to identify seta MN1, although it is most likely present and obscured by the additional setae. *Maxilla* (Figs 7–8). Cardo with one short hair-like seta (MX1) and one short additional seta on ventral surface; stipes with two short hair-like setae (MX2, MX3) and two pores (MXa, MXb) on ventroexternal margin, one very short seta (MX4) ventrally near base of lacinia, and one short straight hair-like seta (MX5) and one very short curved spine-like seta (MX6) dorsally at base of lacinia; proximal segment of GA with one short hair-like seta (MX7) on ventral surface and one short hair-like additional seta on dorsal surface; distal segment of GA with one short hair-like seta (MX8) on dorsoproximal surface, two pores (MXd, MXh) on ventroproximal surface, and one short hair-like seta (MX9) and two minute additional setae near apex; PPF with one short hair-like seta (MX10) on ventral margin; MP1 with one pore (MXe) and one minute seta (MX13) on ventroproximal portion, and one pore (MXf) on dorsodistal portion; MP2 with one pore (MXg) on ventroproximal portion and two short hair-like setae (MX11, MX12) on dorsodistal and ventrodistal portions respectively; MP3 with one pore (MXj) dorsally at about mid length, one short hair-like seta (MX14) ventrally near apex, and several minute pore-like additional structures both on dorsal and ventral surface. *Labium* (Figs 9–10). Prementum with three short hair-like setae (LA3, LA4, LA5) and one pore (LAa) on dorsodistal surface, one short hair-like seta (LA2) and one minute additional pore on ventrodistal surface, and one minute seta (LA1) on ventroproximal surface; LP1 with one minute seta (LA9) on ventroproximal portion and one pore (LAb) on ventrointernal margin at about mid length; LP2 with one pore (LAc) on external margin at about mid length, one short hair-like seta (LA12) ventrally near apex, and several minute pore-like additional structures both on dorsal and ventral surface. *Thorax*. Surface of thoracic terga with numerous hair-like setae. *Legs* (Figs 11–12). Anterior surface of CO with six very short spine-like setae (CO1, CO2, CO3, CO4, CO5, CO17) and two short hair-like setae (CO7, CO18) on proximal portion, one long hair-like seta (CO6) and one pore (COa) on medial portion, and three short hair-like setae (CO8, CO9, CO10) on distal portion; posterior surface of CO with four very short spine-like setae (CO13, CO14, CO15, CO16) on proximal portion, one short hair-like seta (CO12) on medial portion, and one short spine-like seta (CO11) and one pore (COd) on distal portion; anterior surface of TR with one short hair-like seta (TR1) on dorsal margin, one long (TR4) and one short (TR3) hair-like setae on ventrodistal margin, five pores (TRa, TRb, TRc, TRd, TRe) on central portion, and one short hair-like seta (TR7) and one additional pore on ventral margin; posterior surface of TR with two short hair-like setae (TR5, TR6) and two pores (TRf, TRg) on distal margin; anterior surface of FE with one short spine-like seta (FE1) and one pore (FEb) on proximal portion and two short spine-like setae (FE2, FE3) on distal portion; posterior surface of FE with three short spine-like setae (FE4, FE5, FE6) on distal portion; anterior surface of TI with one long hair-like seta (TI1) on proximal portion and three short spine-like setae (TI2, TI3, TI4) on distal portion; posterior surface of TI with one pore (TIa) on central portion, and two short spine-like setae (TI5, TI6) and one short hair-like seta (TI7) on distal portion; anterior surface of TA with one pore (TAa) at about mid length, and three short spine-like setae (TA2, TA3, TA4), one minute seta (TA7), and two pores (TAc, TAd) on distal portion; posterior surface of TA with one short spine-like seta (TA1) at mid length, and two short spine-like setae (TA5, TA6) and three pores (TAb, TAe, TAf) on distal portion; anterior surface of PT with one short spine-like seta (PT1) on basoventral portion; posterior surface of PT with one short spine-like seta (PT2) on basoventral portion. *Abdomen*. Segments I–IX with several mostly hair-like setae on dorsal and ventral surfaces; tracheal gills with one long hair-like seta at tip and one relatively shorter hair-like seta near tip, other setae (if present) obscured by spinulae; segment X (Fig. 13) with numerous spine-like setae and four pores on ventral surface; terminal hooks (Figs 13–15) with two pores on ventral margin at about mid length.

***Instar II***

As instar I except for the following features:

Color. Frontoclypeus, region of parietals posterior to frontoclypeus, and neck region brown, rest of cephalic capsule testaceous to light brown; mandible brown, rest of head appendages testaceous to light brown; protergite with a large subtriangular brown macula on anteromedial region.

Body. Measurements and ratios that characterize the body shape are shown in Table 1.

Head. *Cephalic capsule*. Occipital suture weakly delimited; rugosity on neck restricted to area of occipital suture; egg bursters absent. *Antenna*. About as long as HW; A2 the longest, A3 slightly shorter than A2, A4 slightly shorter than A3. *Maxilla*. MP1 and MP2 subequal in length, somewhat shorter than MP3. *Labium*. LP1 somewhat longer than LP2.

Thorax. Ventral sclerite of prothorax lacking sagittal line. *Legs*. Posterior claw shorter than anterior claw on all legs.

Abdomen. Tracheal gills of segment I almost devoid of spinulae, those of segment II with few spinulae.

Chaetotaxy. Cardo with 7–10 short hair-like secondary setae; secondary leg setation detailed in Table 2.

***Instar III*** (Fig. 16)

As instar II except for the following features:

Color. Same pattern but darker and more obvious in general.

Body. Measurements and ratios that characterize the body shape are shown in Table 1.

Head (Fig. 16). *Antenna*. Somewhat shorter than HW. *Maxilla*. MP1 longest, MP2 shortest, MP3 slightly longer than MP2. *Labium*. LP1 considerably longer than LP2.

Abdomen. Spiracles present on dorsolateral margin of segments I–III.

Chaetotaxy. Cardo with 8–10 short hair-like secondary setae; secondary leg setation detailed in Table 2.

**Table 2.** Number and position of secondary setae on the legs of larvae of *Dineutus sinuosipennis* Laporte, 1840. Numbers between slash marks refer to pro-, meso-, and metathoracic legs, respectively. A: anterior; D: dorsal; P: posterior; V: ventral.

**Segment**	**Position**	**Instar II (n = 3)**	**Instar III (n = 3)**
Coxa	A+AD	13–16 / 14–15 / 11–18	20–26 / 19–25 / 22–27
	PD	5–9 / 6–7 / 6–8	7–9 / 6–11 / 7–9
Femur	AV	4–7 / 6–7 / 6–8	4–5 / 6–8 / 8–9
Tibia	AV	2–3 / 3–4 / 4–5	3–4 / 4–5 / 4–5
Tarsus	PV	5–6 / 5–6 / 6–7	5–6 / 6–7 / 7

### Larval habitat

**Figures 17–18.**
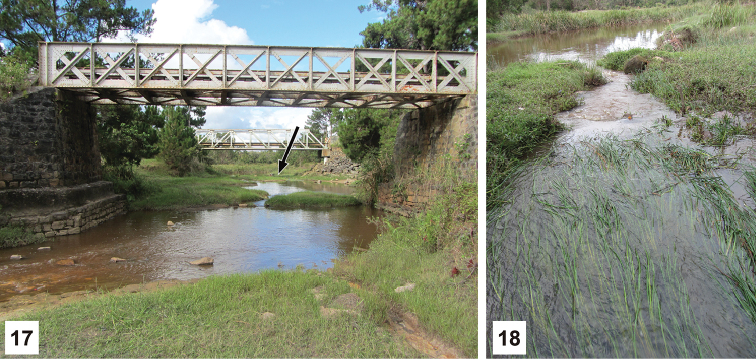
Betsabora River, Madagascar, where *D. sinuosipennis* was collected. **17** General habitat, arrow indicates location of rivulet **18** Specific habitat where larvae were collected within the rivulet.

Specimens of *D. sinuosipennis* were collected on November 28th 2014 in the Betsabora River at crossing of Route National 2, Madagascar (Figs 17–18). The locality is situated near Antsampanana Village, 6 km W of Moramanga (18.9247S, 48.1828E). This is part of the eastern Escarpment and lies at an altitude of 900 m. The main body of the river was relatively slow moving with brown, murky water, and a stone and mud bottom (Fig. 17). Marginal vegetation consisted of reeds in some areas, with most margins having only grass and sparse vegetation. Adults of *D. sinuosipennis* were found in the main river-body near the margins. The river also had a small rivulet that left the main river-body, flowing swiftly over reeds before rejoining it a short distance later (Fig. 18). Larvae of *D. sinuosipennis* were discovered amongst the submerged reeds within this more swiftly flowing rivulet. One dip of the aquatic net within the reeds produced several larvae, while attempts to collect at the margins of the rivulet and the main river-body did not. Nearly 80 larvae of all three instars were collected from this habitat (Fig. 18).

Very few other aquatic beetle adults (notably no adult *D. sinuosipennis*) but many juvenile aquatic insects (such as odonates) were found in this habitat, and it was the only place where larval *D. sinuosipennis* were collected in abundance during the 2014 Madagascar expedition. Given the lack of adult *D. sinuosipennis*, the abundance of its larval form, and lack of similar numbers of larval specimens encountered elsewhere, such fast-flowing, reedy habitats may represent a preferred larval habitat. This type of habitat is easily over-looked, and may explain why ample larval specimens of one of Madagascar’s most common *Dineutus* species have not been previously collected or described.

### Key to larvae of the tribes of Gyrininae and genera of *Dineutini*

The key was constructed for all instars. Although larvae of *Enhydrus* were examined only as instar I, the selected character likely applies also to later instars. Larvae of *Porrorhynchus* are unknown and could not be included. It is likely that larvae of this genus key to the closely related genus *Dineutus*. Known distribution of *Porrorhynchus* comprises southeast Asia from southern China to the greater Sunda Islands and west to Myanmar; also in Sri Lanka (Gustafson and Miller 2017).

**Table:** 

1	Anterior margin of frontoclypeus lacking teeth (Fig. 21)	**Orectochilini**
–	Anterior margin of frontoclypeus with more or less well developed teeth (Figs 22–26)	**2**
2	Stipes with a series of small hook-like setae on internal margin (Fig. 27)	**Gyrinini**
–	Stipes lacking a series of small hook-like setae on internal margin (Figs 28–29) (Dineutini)	**3**
3	Lacinia with posterior margin dentate and apex not deeply indented (Fig. 28) (*Macrogyrus*)	**4**
–	Lacinia with posterior margin not dentate and apex deeply indented (Fig. 29)	**5**
4	Neck constriction present (Fig. 19)	**Subgenus Andogyrus**
–	Neck constriction absent (Fig. 20)	**Subgenus Macrogyrus**
5	Tracheal gills devoid of spinulae (Fig. 30)	***Enhydrus***
–	Tracheal gills bearing two rows of long spinulae (Fig. 31)	***Dineutus***

**Figures 19–31.**
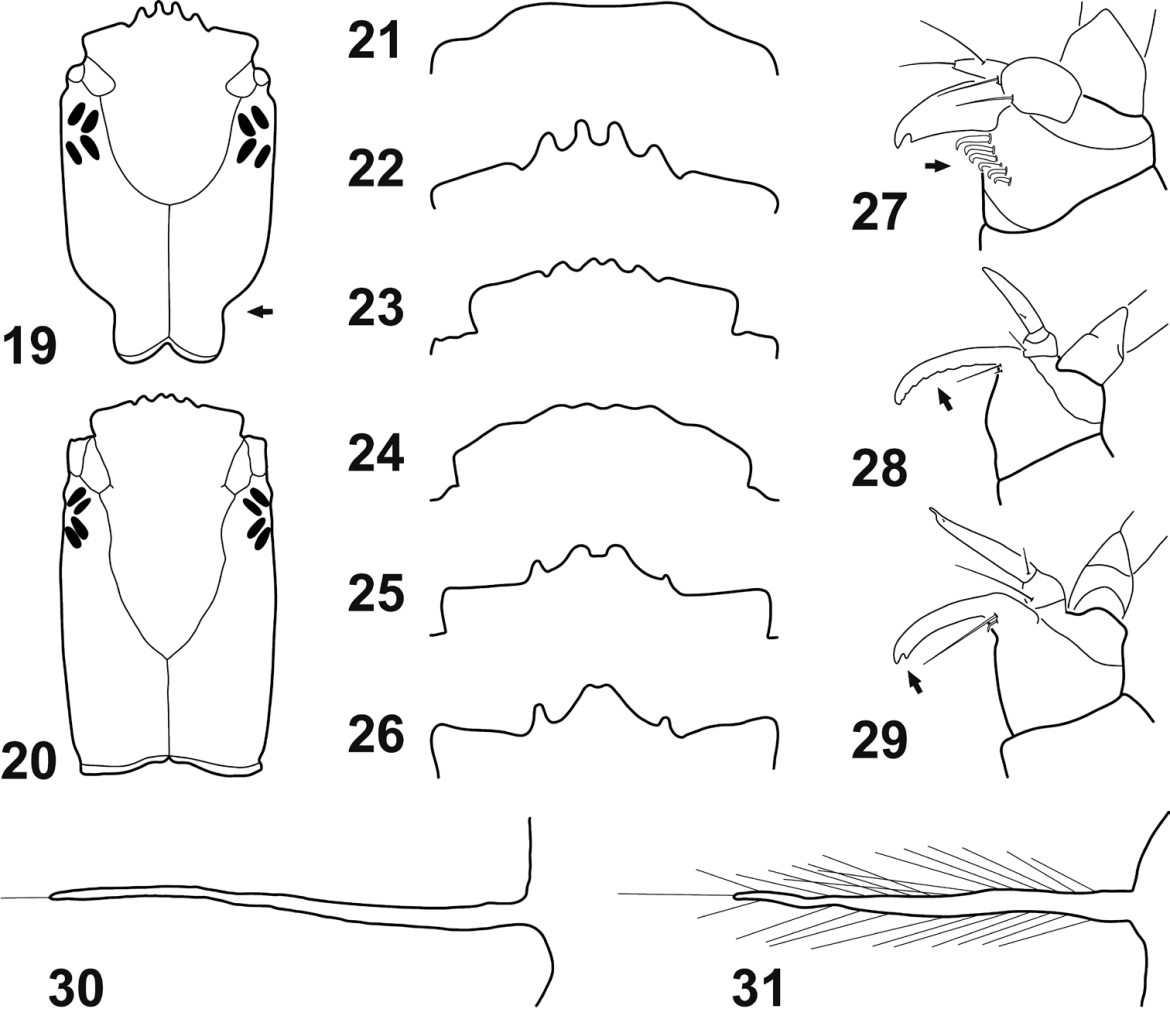
Larval structures (instar I). **19–20** Cephalic capsule, dorsal view **21–26** Anterior margin of frontoclypeus, dorsal view **27–29** Stipes, lacinia and galea, dorsal view **30–31** Tracheal gills, dorsal view **19** Macrogyrus (Andogyrus) seriatopunctatus (Régimbart, 1883) **20** Macrogyrus (Macrogyrus) oblongus (Boisduval, 1835) **21** *Gyretes* sp. **22** M. (A.) seriatopunctatus **23** M. (M.) oblongus **24** *Enhydrus sulcatus* (Wiedemann, 1821) **25** *Dineutus sinuosipennis* Laporte, 1840 **26** *Dineutus* sp. (specimen from Bayfield County, Wisconsin, USA) **27** *Gyrinus monrosi* Mouchamps, 1957 **28** M. (M.) oblongus **29** *D. sinuosipennis* **30** *E. sulcatus* **31** *D. sinuosipennis*.

## Discussion

The description provided here adds *Dineutus* to the group of gyrinid genera for which the ground plan of primary chaetotaxy has been described. Considering this description, within the tribe Dineutini only the larvae of the genus *Porrorhynchus* remain unknown.

*Dineutus* larvae bear the characters considered as putative autapomorphies of Gyrinidae by previous authors (Beutel and Roughley 1994, 2005, Archangelsky and Michat 2007, Michat and Gustafson 2016, Michat et al. 2016): a less sclerotized body, egg bursters located on the parietal, one additional sensorial plate on the third antennomere, a well-developed cardo and lacinia, a completely divided prementum, lateral abdominal tracheal gills, and four terminal hooks on the pygopod. They also share with larvae of the other known Dineutini genera these putative synapomorphies: presence of numerous minute pore-like additional structures on the ultimate maxillary and labial palpomeres, submedial position of the primary seta CO12 on the coxa, and absence of the primary seta TR2 on the trochanter.

Compared to those of the other known Dineutini genera, larvae of *Dineutus* can be distinguished by the lacinia not dentate on posterior margin (= *Enhydrus*, dentate in *Macrogyrus*); tracheal gills plumose (= *Macrogyrus*, not plumose in *Enhydrus*); parietal seta PA5 inserted relatively far from setae PA7–9 (closer to PA7–9 in *Enhydrus* and *Macrogyrus*); mandibular pores MNb and MNc inserted relatively far from each other (closer to each other in *Enhydrus* and *Macrogyrus*); and tarsal seta TA1 inserted submedially (inserted distally in *Enhydrus* and *Macrogyrus*). Information regarding these characters in other Dineutini species is scarce, since the larvae of most species are unknown. Therefore, their phylogenetic significance remains to be tested when larvae of more species are studied.
